# Control of household air pollution for child survival: estimates for intervention impacts

**DOI:** 10.1186/1471-2458-13-S3-S8

**Published:** 2013-09-17

**Authors:** Nigel G Bruce, Mukesh K Dherani, Jai K Das, Kalpana Balakrishnan, Heather Adair-Rohani, Zulfiqar A Bhutta, Dan Pope

**Affiliations:** 1 Department of Public Health and Policy, University of Liverpool, Liverpool L693GB, UK; 2 Department of Public Health and Environment, World Health Organization, via Appia, 1211 Geneva 27, Switzerland; 3 Aga Khan University, Division of Women and Child Health, Aga Khan University, Stadium Road Karachi- 74800, Pakistan; 4 ICMR Centre Department of Environmental Health Engineering, Sri Ramachandra University, No.1, Ramachandra Nagar, Porur, Chennai-600116, India

## Abstract

**Background:**

Exposure to household air pollution (HAP) from cooking with solid fuels affects 2.8 billion people in developing countries, including children and pregnant women. The aim of this review is to propose intervention estimates for child survival outcomes linked to HAP.

**Methods:**

Systematic reviews with meta-analysis were conducted for ages 0-59 months, for child pneumonia, adverse pregnancy outcomes, stunting and all-cause mortality. Evidence for each outcome was assessed against Bradford-Hill viewpoints, and GRADE used for certainty about intervention effect size for which all odds ratios (OR) are presented as protective effects.

**Results:**

Reviews found evidence linking HAP exposure with child ALRI, low birth weight (LBW), stillbirth, preterm birth, stunting and all-cause mortality. Most studies were observational and rated low/very low in GRADE despite strong causal evidence for some outcomes; only one randomised trial was eligible.Intervention effect (OR) estimates of 0.64 (95% CI: 0.55, 0.75) for ALRI, 0.71 (0.65, 0.79) for LBW and 0.66 (0.54, 0.81) for stillbirth are proposed, specific outcomes for which causal evidence was sufficient. Exposure-response evidence suggests this is a conservative estimate for ALRI risk reduction expected with sustained, low exposure. Statistically significant protective ORs were also found for stunting [OR=0.79 (0.70, 0.89)], and in one study of pre-term birth [OR=0.70 (0.54, 0.90)], indicating these outcomes would also likely be reduced. Five studies of all-cause mortality had an OR of 0.79 (0.70, 0.89), but heterogenity precludes a reliable estimate for mortality impact. Although interventions including clean fuels and improved solid fuel stoves are available and can deliver low exposure levels, significant challenges remain in achieving sustained use at scale among low-income households.

**Conclusions:**

Reducing exposure to HAP could substantially reduce the risk of several child survival outcomes, including fatal pneumonia, and the proposed effects could be achieved by interventions delivering low exposures. Larger impacts are anticipated if WHO air quality guidelines are met. To achieve these benefits, clean fuels should be adopted where possible, and for other households the most effective solid fuel stoves promoted. To strengthen evidence, new studies with thorough exposure assessment are required, along with evaluation of the longer-term acceptance and impacts of interventions.

## Introduction

Household air pollution (HAP) from solid fuels (wood, dung, crop residues, charcoal and coal) used in simple stoves for cooking and heating, is recognized as a risk factor for several health outcomes with important consequences for child survival, including pneumonia [1] and low birth weight and stillbirth [2], in addition to a number of major non-communicable disease outcomes in adults [3,4].

Solid fuels were used by around 2.8 billion people in 2010 [5], a number which has changed little since 1980 due to global population increase, and is closely associated with poverty and high child mortality. Studies consistently show that exposure levels are very high, far exceeding WHO air quality guideline (AQG) levels for small particulate matter (PM_2.5_), and young children and women, including during pregnancy, are most at risk [6,7]. These factors imply that, if substantial intervention effects can be demonstrated, the removal of HAP exposure could bring large benefits for child survival.

Interventions for reducing HAP exposure include improved solid fuel stoves and clean fuels. Both present challenges for sustainable adoption at scale among the low income populations at risk [8]. For solid fuel stoves the key issues are achieving emissions low enough to deliver health benefits, as well as ensuring acceptability, sustained use, and affordability. Very low exposure would be assured if households used clean fuels such as LPG and electricity exclusively, but affordability and reliable supply remain key barriers. Recent initiatives to increase global access to clean household energy, including the UN Foundation Global Alliance for Clean Cookstoves [9], and the UN’s Sustainable Energy for All [10], are now starting to address these issues in a coordinated way. More concerted action on and investment in technology development, stove standards [11], programme delivery, and evaluation can be expected over the next few years.

The objective of this review is to systematically review the evidence on HAP and child survival outcomes and to propose intervention impact estimates that would be suitable for the Lives Saved Tool [12]. While detailed assessment of intervention options, performance and policy for achieving sustained adoption is also important, these topics are beyond the scope of this review. These issues have been discussed elsewhere [8], and extensive review work is currently underway for new WHO Guidelines on household fuel combustion [13].

The scope of the review is as follows. For health outcomes, those listed by Walker et al [14] for children under 5 years and known or suspected to be linked to HAP were included, namely ALRI (pneumonia, including severe and fatal), low birth weight, pre-term birth, stillbirth, stunting, and all-cause mortality. Geographical coverage is global where the use of solid fuels for cooking has been studied. Although there is evidence that kerosene used in simple stoves and lamps is also highly polluting and a health risk for some of these outcomes [15], this was outside the scope of the review, except to note studies which included kerosene within the ‘clean fuel’ category.

## Methods

### Exposure assessment

A database of household energy, managed by WHO, draws information from nationally representative surveys including DHS, MICS, LSMS, World Health Survey, and national censuses on the primary fuel used for cooking. To date, data from some 586 surveys have been collated for 155 countries, spanning 1974 to 2010 [16,17]. Surveys obtain information on specific fuel types, but the main indicator used to assess exposure to household air pollution is use of solid fuel for cooking, and is available stratified by urban and rural settings within countries. It is recognized that this is a relatively crude measure of exposure, and one approach for improving this through modeling is considered further in the Discussion.

### Reviews of health risks

We have previously published reviews for pneumonia [1] and adverse pregnancy outcomes (LBW, stillbirth and pre-term birth) [2]. The current report updates these published reviews using comparable methods, and includes new reviews conducted for stunting and all-cause mortality. The reviews cover the period from 1966 to July 2012. Search terms and databases used for all outcomes and study selection flowcharts are presented in Additional file 1; selection included around 10% independent checks of both selected and rejected titles and abstracts. Full duplicate data extraction and quality assessment using a modified Newcastle-Ottawa scale was conducted, with disagreements resolved by a third researcher. All study designs were eligible, but studies were excluded if outcome definitions were unclear (e.g. no differentiation between upper and lower respiratory infections). Analysis was carried out in RevMan (version 5.1), and pooling of studies used generic inverse variance weighting with fixed effects in the absence of statistical heterogeneity (I-squared < 10%), otherwise random effects meta-analysis was conducted. For outcomes with more than 4 studies, publication bias was assessed through statistical funnel plot asymmetry with Begg’s and Eggar’s tests using Stata Version 10 [18].

**Figure 1:**
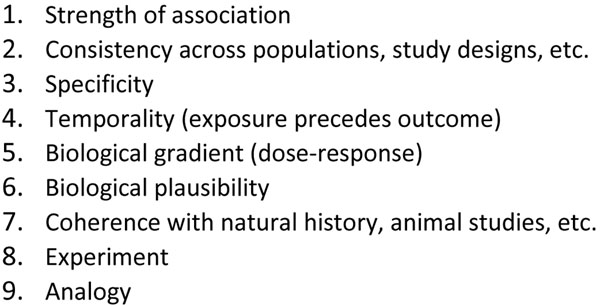
Bradford-Hill viewpoints on assessing causation in environmental health

Assessment of the evidence for each of the outcomes using GRADE [19] was carried out by JD, DP, MD and NB in a 2-day workshop in July 2012. Recent debate on the application of GRADE in the assessment of studies of public health interventions suggests modifications may be needed for more appropriate rating of this evidence [20-22]. In this regard, it is useful to make a distinction between (i) the question of whether associations reported here are causal, for which Bradford-Hill viewpoints for distinguishing causation from association in environmental epidemiology (Figure 1) [23] are referred to, and (ii) the strength of evidence for the intervention effect size, for which GRADE has been used. While these assessments have much in common, it is quite possible to have strong evidence of causal associations between HAP exposure and one or more of the disease outcomes (and by implication that reducing exposure will reduce the risk of that disease), but rather weaker evidence as to the precise size of the effect of an intervention. As this debate on modifying GRADE for public health is ongoing, we have first referred to the Bradford-Hill viewpoints and then applied standard GRADE methodology, and consider the implications further in the Discussion.

The majority of studies available are observational and report risks of high vs. low exposure, although in the context of this review we are interested in estimating the potential preventive impact of interventions. Only the single eligible RCT (‘RESPIRE’) has reported results in this way [24]. In order to achieve consistency across studies while avoiding potentially misleading changes to the existing published results, the Forest plots are presented as increased risk associated with higher exposure (as published by the incorporated studies), and for this purpose we have inverted the relative risks reported from our own work [24]. In the GRADE tables (Additional File 2) and reporting of these in the Results, all odds ratios are presented as preventive effects. Issues arising from estimating intervention effects from observational studies are considered further in the Discussion.

A number of intervention studies are available, but most are restricted to measuring impacts on HAP and/or exposure, and do not include health outcomes. This information can, however, provide an indication of the expected health impacts as exposure-response evidence on key child survival outcomes becomes more available and robust. A systematic review of these studies, covering different types of solid fuel stoves and clean fuels, is being conducted for new WHO air quality guidelines [13] and will be reported separately. A selection of these studies showing the range of exposure reductions and post-intervention levels achieved are described in the current report, and related to the limited exposure-response evidence available on child ALRI. The trials that do report health outcomes are discussed in more detail.

## Results

### Exposure

**Figure 2:**
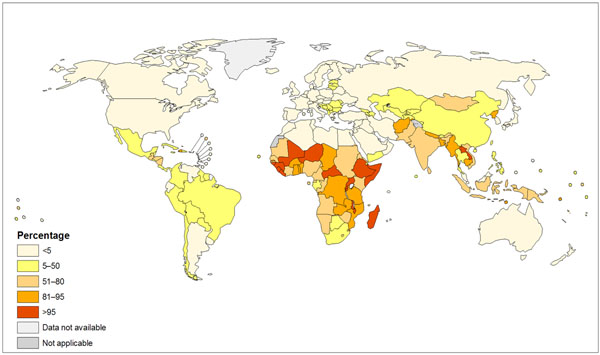
Households using solid fuels as the primary cooking fuel, by WHO region, 2010. Source: WHO Global Health Observatory: http://gamapserver.who.int/mapLibrary/Files/Maps/Global_iap_2010_total.png

In 2010, 41% of global households still relied primarily on solid fuels for cooking, a figure which has fallen from 62% in 1980, but because of population increases the actual number has remained steady at around 2.8 billion people [17]. Solid fuel use closely mirrors poverty and child mortality (Figure 2), and remains highly prevalent in South Asia and Africa, including more than 90% of homes in rural areas of many countries of Sub-Saharan Africa [5]. Regional trends show that, while the proportion of homes cooking with solid fuels has declined by around 25-35% in most regions since 1980, in Africa the decline has been only 10%, from 87% to 77% [17].

### Child health outcomes

#### Child ALRI

A total of 26 eligible studies (28 estimates) were found for child ALRI, including all non-fatal ALRI (severity not defined), severe ALRI, and fatal ALRI outcomes (reported separately below). The pooled OR for all studies was 1.73 (95% CI=1.47, 2.03), and although there was evidence of publication bias (Egger’s test p=0.046), this may be partly the result of larger effects for more severe and fatal outcomes being reported in smaller studies.

#### All non-fatal ALRI (severity not defined)

**Figure 3:**
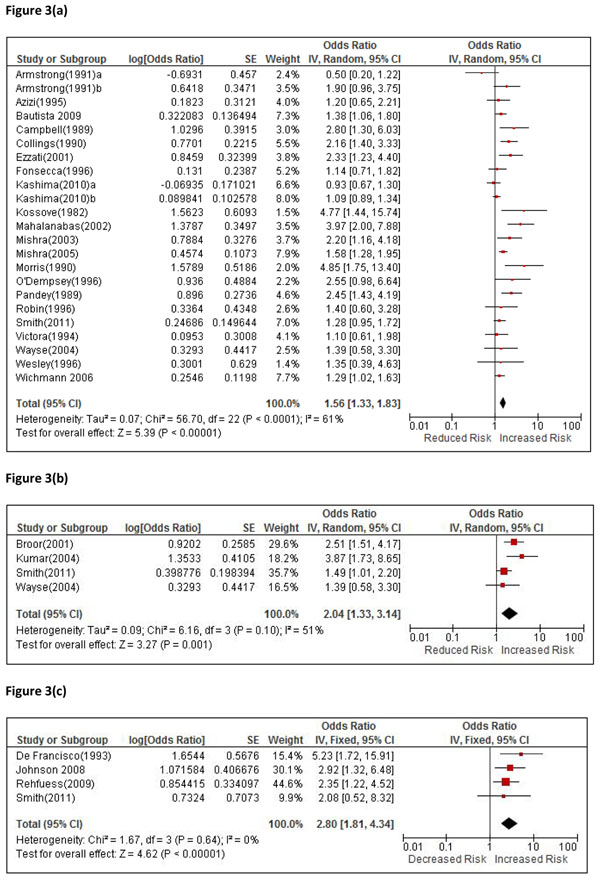
Forest plots All individual study results are presented as originally published (risk of outcomes for high exposure vs. low exposure) with the exception of the RESPIRE trial, for which relative risks have been inverted for consistency. Results in GRADE tables (Additional File 2) are all presented as protective effects. Figure 3(a): Forest plot for 20 studies of non-fatal ALRI, where severity is not defined Figure 3(b): Forest plot for 4 studies of severe ALRI Figure 3(c): Forest plot for 4 studies of fatal ALRI

A total of 21 studies reported on non-fatal ALRI, without defining severity, including one RCT [24], 4 cross-sectional [25-28], 11 case-control [29-39] and 5 cohort studies [40-44], summarized by study design in Additional File 3, Table 1(a). The RCT by Hanna et al was not eligible as the outcome measures did not allow distinction of upper and lower respiratory infections [45]. The funnel plot suggests possible publication bias (Additional File 4, Figure 1), but Begg’s (p=0.56) and Eggar’s (p=0.091) tests were non-significant. There was significant heterogeneity (I^2^ = 61%, p<0.0001), and the pooled OR was 1.56 (1.33, 1.83), p<0.0001, Figure 3(a). Some duplication of cases in the RESPIRE trial occurs with the severe ALRI analysis below, but exclusion of this study results in a small increase in the effect estimate to 1.59 (1.34, 1.89). A concern with several studies in this review is inclusion of kerosene in the ‘unexposed’ group, which involves just the Indonesian DHS study for this outcome [25]: the pooled OR following exclusion of this study was 1.66 (1.41, 1.97) with I^2^ reduced to 52% (p=0.003). The GRADE assessment for the RCT was rated ‘moderate’ with a RR of 0·78 (0·59, 1·06), for which a statistically significant exposure-response relationship was also noted, while for the observational studies the rating was ‘very low’, with a protective OR of 0.63 (0.53, 0.75), Additional File 2, Table 1(a).

#### Severe ALRI

Four studies, including one RCT [24] and three case-control studies [46-48] reported on severe ALRI, Additional File 3, Table 1(b). Heterogeneity was of borderline significance (I^2^=51%, p=0.10), and the pooled OR was 2.04 (1.33, 3.14) p=0.001, Figure 3(b). GRADE assessment for the RCT was ‘high’ with RR of 0·67 (0·45–0·98), again with significant exposure-response findings for this outcome, while the three observational studies were rated ‘low’ with a protective OR of 0.40 (0.25, 0.67), Additional File 2, Table 1(b).

#### Fatal ALRI

Four studies, including one RCT (9 events) [24], and three observational studies reported risk of fatal pneumonia [49-51], Additional File 3, Table 1(c). There was no evidence of statistical heterogeneity (I^2^=0), and the pooled OR was 2.80 (1.81, 4.34) p<0.0001, Figure 3(c). GRADE assessment rated the RCT as ‘high’ with a RR of 0.48 (0.12, 1.91) and the three observational studies as ‘low’, with a protective OR of 0.34 (0.22, 0.55), Additional File 2, Table 1(c).

#### Low birth weight

**Figure 4:**
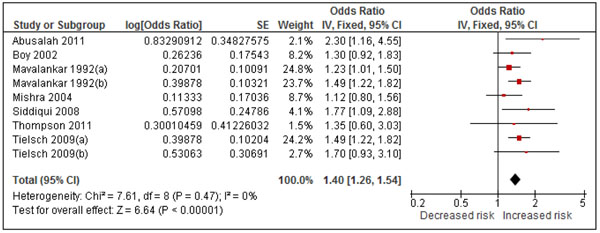
Forest plot for 7 studies of low birth weight. Those marked (b) are separate estimates for pre-term IUGR

Seven studies, including one RCT (analyzed per protocol) [52] and six observational studies [53-58] reported on the risk of low birth weight (<2500 gm at term), Additional File 3, Table 2. Two studies provided separate, independent estimates for pre-term IUGR and term LBW [54,57]. There was no evidence of statistical heterogeneity (I^2^=0), nor of publication bias (Begg’s p=0.348, Eggar’s p=0.356). The pooled OR was 1.40 (1.26, 1.54) p<0.0001 for all births, Figure 4, and 1.36 [1.20, 1.54] for term births, while for pre-term the OR was slightly higher at 1.51 [1.25, 1.83]. Sensitivity analysis restricted to four studies carrying out adequate adjustment [52,56-58] had a larger effect for term LBW of 1.57 (1.33, 1.86). GRADE assessment rated the RCT as ‘moderate’ with a RR of 0.74 (0.33-1.66), while the observational studies were rated ‘low’ with a protective OR of 0.71 (0.64, 0.79), Additional File 4, Table 2.

#### Stillbirth

**Figure 5:**
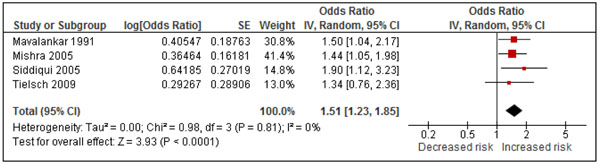
Forest plot for 4 studies of stillbirths

No new eligible studies of stillbirth were identified in the review update, Additional File 3, Table 3. The RCT by Hanna et al included stillbirth but was not eligible as this outcome was combined with infant mortality and miscarriage [45]. The Indonesian DHS-based study by Kashima et al was excluded as stillbirth was combined with miscarriage and abortion [25]. Among the four observational studies included there was no evidence of statistical heterogeneity (I^2^=0%), and the pooled OR was 1.51 (1.23, 1.85), Figure 5; GRADE assessment was ‘low’, with a protective OR of 0.66 (0.54, 0.81), Additional File 4, Table 3.

#### Pre-term birth

Only one study was identified with an estimate for pre-term birth [57]. This reported an OR of 1.43 (1.11, 1.84), Additional File 3, Table 4, and was rated ‘low’ in GRADE assessment, with a protective OR of 0.70 (0.54, 0.90), Additional File 4, Table 2.

#### Stunting

**Figure 6:**
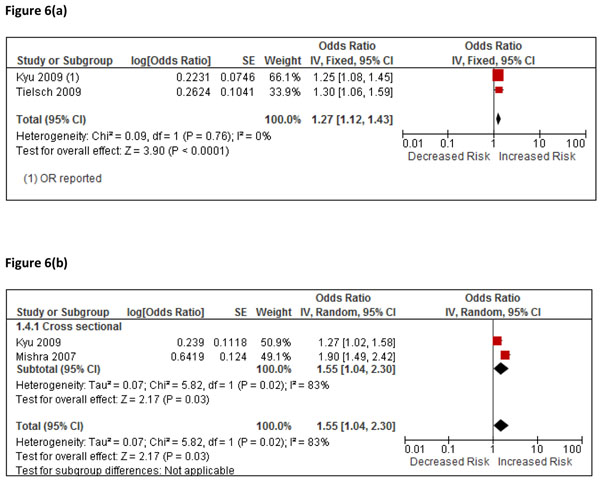
(a): Forest plot for 2 studies of moderate stunting Figure 6(b): Forest plot for 2 studies of severe stunting

Four studies reported on the risk of stunting, two for moderate stunting (-3 SD ≤ Z to -2 SD) [57,59], and two for severe stunting (Z < -3 SD) [59,60], Additional File 3, Table 5. One was based on an Indian cohort study [57], the others cross-sectional using DHS data for 7 countries [59], and India [60]; all studies provided adjusted estimates. For moderate stunting, there was no evidence of statistical heterogeneity (I^2^=0%), and the pooled OR was 1.27 (1.12, 1.43) p<0.0001, Figure 6(a). GRADE assessment was ‘low’ with a protective OR of 0.79 (0.70, 0.89), Additional File 2, Table 5. For severe stunting, there was evidence of significant heterogeneity (I^2^=83%, p=0.02), the pooled OR was 1.55 (1.04, 2.30), Figure 6(b), and GRADE assessment was rated ‘very low’ with a protective OR of 0.64 (0.43, 0.96), Additional File 2, Table 5. The study by Kyu et al combined DHS data from 7 countries with very different socio-economic conditions, including percentage use of solid fuels and children’s nutritional status [59].

#### All-cause mortality

**Figure 7:**
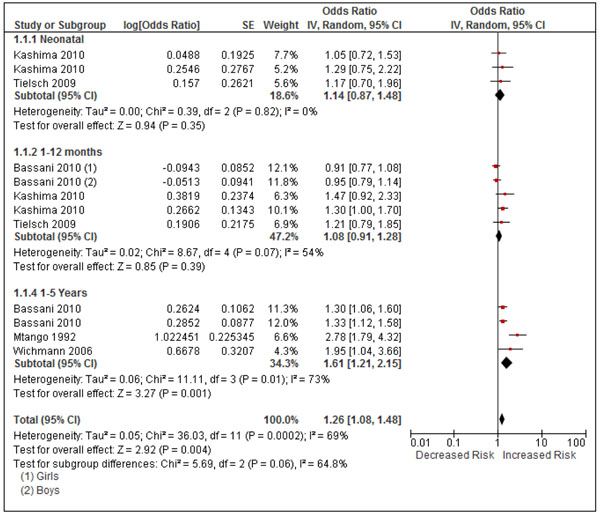
Forest plot for 5 studies of all-cause mortality, stratified by age group. Note: The estimate from Tielsch et al (2009) in the 1-12 month age group is for 0-6 months and includes the neonatal deaths: see text for further explanation.

Five studies provided estimates for all-cause mortality, two case control [61,62], two cross-sectional based on the DHS [25,63], and one cohort study [57], spanning several age groups, Additional File 3, Table 6. All five studies provide adjusted estimates. The RCT by Hanna et al assessed infant mortality, but has not been included for the reasons given in the Discussion [45]. There was significant heterogeneity across all studies (I^2^=72%, p<0.0001), in addition there was possible evidence of publication bias [(Begg’s test p=0.466; Eggar’s test p=0.081, Additional File 4, Figure 2. The pooled OR was 1.27 (1.07, 1.50) suggesting an overall significant impact on mortality (not shown); stratified analysis with all study estimates is shown in Figure 7. Two studies provided estimates for neonatal mortality, with an I^2^ =0% and a non-significant pooled OR of 1.14 (0.87, 1.48) [25,57], but both included kerosene in the ‘unexposed’ group. For the 1-12 month age group, three studies are included, although Tielsch et al focused on 0-6 months, and hence also covers the neonatal period. There was evidence of statistical heterogeneity (I^2^=54%, p=0.07), and the pooled OR of 1.08 (0.91, 1.28) was non-significant. All three studies included kerosene in the ‘unexposed’ group. Exclusion of the Tielsch et al estimate made little difference to the pooled estimate.

The largest effect was seen in the 1-5 year age group, where there was significant statistical heterogeneity (I^2^=73%, p=0.01) with a significant pooled OR of 1.61 (1.21, 2.15). Only one of these studies included kerosene in the ‘unexposed’ group [62], while Wichmann et al included kerosene in the ‘exposed’ group [63].

GRADE assessment rated the evidence as ‘very low’, due to inconsistency and possible publication bias. The protective OR was 0.79 (0.67, 0.93), Additional File 2, Table 6. While assessment within age groups would be possible, this is unlikely to change the overall rating as significant heterogeneity was seen in the one age group with a significantly elevated risk (1-5 years).

#### Summary of effect estimates

Based on the findings of the review, consideration of the Bradford-Hill viewpoints, GRADE assessments and application of the Rules recommended by CHERG [14], the following effect estimates are proposed.

#### ALRI

For ALRI, our earlier published review reported an OR of 1.78 (1.45, 2.18) [1], and the update has made little difference. The volume of evidence and reference to the Bradford-Hill viewpoints (including intervention impacts and exposure-response relationships), suggest a high level of confidence concerning causality. In a 2011 review, Po et al reported a much larger effect of 3.53 (1.94, 6.43) for child ARI but there were some important methodological differences [64]. Thus, Po et al grouped studies reporting ARI and ALRI, whereas we excluded studies not distinguishing upper and lower ARI and included others not cited by Po et al. We also did not include two studies cited by Po et al that reported surprisingly large estimates, the first as the unadjusted OR of 32.6 was stated as non-significant in adjusted analysis but not provided [65], while for the second study the OR for children of 7.98 (quoted by Po et al) used comparison with an ‘unexposed’ group of ‘other members of the family’ [66].

While the results of the RESPIRE trial are important, it would be inappropriate to base the effect estimate here on this single study. Furthermore, since the intervention group exposure remained high (relative to the WHO air quality guideline level), larger effects may be seen with community-wide use of clean fuels or low emission biomass stoves. Indeed, the exposure-response results from RESPIRE show that exposures below the intervention group mean were associated with considerably lower risk for all ALRI and severe ALRI [24]. These combined intention-to-treat and exposure-response findings from RESPIRE are consistent with the observational studies, both in terms of effect size and the greater effect on more severe outcomes.

These protective effect estimates for severe and fatal ALRI are relatively large, and require confirmation through further intervention studies before being considered as a basis for LiST. For the purpose of providing a realistic but conservative estimate given the current state of intervention-based evidence, it is proposed Rule 4 be used, taking the smallest of the pooled estimates, that for all non-fatal ALRI with severity not defined: 0.64 (0.55, 0.75). It is expected, however, that sustained, low exposure close to the WHO AQG level (annual mean PM2.5 of 10 µg/m^3^) would result in larger effects, especially for severe and fatal pneumonia. In this respect, it is noted that the recently published Global Burden of Disease 2010 comparative risk assessment used a larger relative risk for child ALRI than proposed here, but this was in the context of describing total population attributable risk with a counterfactual scenario of all homes using clean fuels and a mean PM2.5 exposure of less than 10 µg/m^3 ^[4].

### Adverse pregnancy outcomes

For low birth weight, the high degree of consistency across studies and the strong supportive evidence from other sources of combustion and animal studies [2] suggest a causal effect. Using Rule 6, with GRADE rating (moderate for the RCT and low for the observational studies), an intervention effect estimate of 0.71 (0.65, 0.79) is proposed. Evidence for stillbirth is less strong, but the consistency, supporting evidence from ambient air pollution and biological plausibility [2] are judged sufficient to support an intervention estimate using Rule 3 of 0.66 (0.54, 0.81). For pre-term birth, more studies are needed to strengthen causal inference, and we do not propose an intervention effect estimate at this time.

#### Stunting

For stunting, two pooled estimates are available for both moderate and severe stunting, albeit data are drawn from seven countries in one study contributing to both outcomes [59], and further evidence is required to increase confidence about a causal effect. Accordingly, we do not propose an intervention effect estimate at this time.

#### All-cause mortality

The evidence on all-cause mortality suggests HAP exposure increases risk, with a significant pooled estimate across the whole under 5 year age group being 1.27 (1.07, 1.50). However, this is a heterogeneous set of studies, and a consistent effect across the different stages of the first 5 years of life, including the neonatal and post-neonatal periods, seems unlikely. Too few studies are available within each age group to provide risk estimates with any confidence. A causal effect on all-cause mortality is, however, highly likely given the strength of evidence on ALRI (including severe/fatal) as well as for adverse pregnancy outcomes, in particular low birth weight. We do not consider there is sufficient evidence to provide a reliable, direct intervention effect estimate for mortality at this time, and this should be a priority for future research.

## Discussion

Although much of the material in this review has been published previously, additional value derives from (i) updating of existing reviews by the same research group using comparable methods, (ii) stratification of ALRI by severe and fatal outcomes made possible by a number of new studies, (iii) adding stunting and all-cause mortality, (iv) bringing together all of the available child survival relevant outcomes in one review, and (v) the consistent application of GRADE to this evidence which hitherto, has not been attempted. The epidemiological evidence has a number of limitations, including study design bias (e.g. control selection), exposure misclassification, residual confounding, and variable outcome definitions, the implications of which we have discussed previously [1,2,6]. We found little evidence, however, of systematic over-estimation of effects in sensitivity analysis [1,2]. Almost all studies are observational, with only one eligible published RCT testing the impact of an effective intervention.

### Strength of currently available evidence and GRADE assessments

One other recently reported trial from India used a stove with no impact on HAP due to technical limitations and low user valuation [45]. Although several outcomes potentially relevant to this review were included in that study, upper and lower ALRI could not be distinguished (therefore not meeting eligibility criteria) and still births were not presented separately from miscarriages and infant mortality, although infant mortality was reported separately. The reduction in exposure, measured using exhaled CO, was not reported for children under 5 years; that for all children was less than 10% (0.478 ppm), and non-significant. We have not incorporated any of the results from this trial in our review as we feel that the study does not add evidence regarding the impact of HAP reduction on child survival outcomes, and that this was equivalent to conducting an antibiotic or vaccine trial with an ineffective product. Prior to investigating health outcomes, the effectiveness of the candidate stove or cleaner fuel in delivering substantive exposure reductions in everyday use can and should be established, and over a period long enough to be confident that these effects will be sustained. The trial provides a valuable and timely warning of the importance of this, and that assumptions about so-called improved stoves must be tested prior to launching health studies and scaling-up adoption.

The predominance of observational designs means that effect estimates are based mainly on comparisons of rates in ‘high’ and ‘low’ exposure groups, rather than by directly measuring intervention impacts. Although some biases including poor control selection and residual confounding may overestimate effect sizes, inadequate exposure measurement will tend towards underestimation. In most studies, the lower-exposure group was often stated as using cleaner fuels such as LPG and electricity, but it is expected that these homes would have had exposures well above WHO AQG levels (annual mean PM_2.5_ of 10 µg/m^3 ^[67]), for two reasons. First, many developing countries households that state their primary cooking fuel is clean (e.g. LPG) also use solid fuel for some cooking and maybe heating purposes, especially in rural areas [68]. Second, even where households use clean fuels (and perhaps exclusively), they are subject to pollution from neighbours using solid fuels, and in urban areas to annual average levels of ambient air pollution (from household combustion and other sources) that are known to exceed 50-100 µg/m^3^ PM_10_ in many developing country cities [69]. These suppositions are supported by studies reporting average concentrations in the range 50-150 µg/m^3^ (variously measuring PM10, PM4 and PM2.5) among LPG users [70-73]. High ‘background’ exposure in the ‘clean fuels’ groups will under-estimate risk, with effect estimates further biased towards the null by exposure misclassification. This latter point is emphasized by the substantial overlap of exposure distributions between control and intervention groups reported from the RESPIRE study [74].

It is recognised, therefore, that intervention effect estimates based on predominantly observational evidence may not be reliable, and that these are subject to a complex mix of bias. Related to this are the questions of (i) to what level exposure needs to be reduced in order to achieve the intervention impacts proposed here, and (ii) will any useful benefit accrue with lesser exposure reductions? For child ALRI, the RESPIRE exposure-response analysis suggests the function is relatively flat at high levels and the steep fall-off in risk only occurs at a level below that seen in the intervention (chimney stove) group [24]. Although derived from the trial this is effectively an (adjusted) observational analysis, but is consistent in shape with that reported earlier by Ezzati and Kammen [75]. Integration of ALRI risk data for PM2.5 arising from outdoor air pollution (OAP), second-hand smoke (SHS) and HAP (using RESPIRE results) has tended to confirm this conclusion; although details are yet to be published, this methodology was applied in the 2010 Global Burden of Disease (GBD) study comparative risk assessment [4].

In assessing the strength of evidence, we have drawn a distinction between confidence that associations are causal, and confidence about the size of the estimates proposed for intervention impacts. Based on the Bradford Hill viewpoints, we noted there was sufficient evidence for associations with ALRI, LBW and stillbirth being causal, while for others including pre-term birth and stunting further studies are needed for this purpose. GRADE assessments were mostly in the low and very low categories for all of these outcomes (and the single RCT could not be used alone), reflecting the lack of randomized trials and the way in which the current scoring system assesses observational evidence [20]. Furthermore, although observational evidence can be upgraded [21], this was only possible for severe and fatal pneumonia [see Additional File 2, Tables 1(b) and (c)]. Such generally low GRADE assessments imply that effect estimates are not established with confidence and that new evidence may well change these substantially. This may be correct, and for now justifies using the more conservative of the available results. On the other hand, exposure misclassification, the relatively high intervention group mean exposure in RESPIRE, and the emerging exposure-response evidence, suggest that larger impacts can be expected with sustained lower levels of exposure.

### Improved population exposure assessment

The percentage SFU derived from the WHO database provides useful first line estimates of current levels of, and trends in, national and sub-national (e.g. urban/rural) exposure to HAP. Numerous HAP measurement studies, conducted across all WHO regions [76], have also provided unequivocal evidence of very high exposures in solid fuel using households, many times the recommended WHO AQG levels. However, few efforts have been undertaken to estimate population level exposures in quantitative terms.

Work carried out in India [Balakrishnan 2013, under review] and applied in the GBD 2010 comparative risk assessment [4] has been investigating the potential of using empirical HAP measurement studies to model population levels and variations of PM2.5 across India. Community studies in four states were used to collect data on area PM2.5, as well as on household level determinants such as, fuel and stove use, cooking location, etc., variables which are (or can be) collected in national surveys. A predictive model was then generated and applied to variables available from the Indian National Family and Health Survey (2005) to estimate HAP PM2.5 concentrations for each state. Future work will assess the potential for estimating personal exposures for women, children and other family members. While further validation is needed, this methodology offers the possibility of generating more nuanced estimates of HAP and exposure, and while the Indian model cannot be applied directly to other countries, the methodology can.

### Prospects for interventions

We have indicated that, to deliver the estimated benefits reported here, interventions need to reduce HAP to low levels, probably with PM2.5 approaching WHO air quality guidelines although precision on this issue requires further study. Furthermore, these interventions need to be affordable and their use sustained. Addressing these questions in detail is beyond the scope of the current report; systematic reviews of both issues are underway for new WHO guidelines on household fuel combustion [13]. As noted in the Introduction, a range of interventions do exist, including both clean fuels and improved solid fuel stoves. For some socio-economically and geographically defined strata of solid fuel users, well-targeted policy could facilitate a relatively rapid transition to clean fuels such as LPG, though in practice this may be partial use for many with the aim of a more complete substitution over time. For others, however, solid fuels will continue to be relied upon and a key research and policy objective is to determine which solid fuel stove technologies can meet household needs and deliver all or most of the available health benefits.

Studies reporting the impacts of ‘improved’ solid-fuel stoves on HAP (including a mix of cross-sectional, before and after, quasi-experimental and randomized designs) have assessed a range of technologies and shown that many can substantially reduce PM2.5 concentrations in absolute terms, but post-intervention levels remain well above WHO AQG values in the range 100-300 µg/m^3^; see for example Dutta [77], Brandt [78], Chengappa [79] and Chowdhury [80]. Some chimney stoves have been reported to achieve kitchen PM2.5 levels close to the WHO annual IT-1 value of 35 µg/m^3^, for example Terrado [81], but these are the minority. Unfortunately, very few studies of advanced combustion solid fuel stoves (using forced ventilation) in everyday use are available. In summary, among solid-fuel stoves, those with chimneys have resulted in the lowest levels of HAP, but most seem unlikely to deliver very substantial reductions in risk of child ALRI until levels closer to the AQG are reached [24]. The point estimate of a 33% (95% CI: 2-55%) reduction in severe pneumonia from intention-to-treat analysis of the chimney (intervention) stove vs. open fire (control) in the RESPIRE trial is encouraging, but the confidence interval is wide and this requires confirmation [24]. In the absence of exposure-response evidence for other child survival outcomes, it probably should be conservatively assumed that the pneumonia scenario also applies to adverse pregnancy outcomes, child growth and mortality.

To fill these gaps, research is urgently required on the performance and acceptability of more advanced stove designs (with and without chimneys), the role modern fuels can play in advancing access to clean household energy, and on the impacts of these interventions on child health outcomes with studies that incorporate thorough exposure assessment.

## Conclusions

Substantial evidence now exists that HAP increases the risk of a range of outcomes that are important for child survival. The predominance of observational evidence means that effect estimates may not yet be well estimated, but assessment of the various sources of bias suggests that these are unlikely to be exaggerated. Risk reductions of between 29% and 36% for the three outcomes (ALRI, LBW and stillbirth) with the strongest causal evidence are proposed. Interventions that deliver large reductions in HAP with PM2.5 close to WHO AQG levels can be expected to achieve these risk reductions, and quite possibly more, including for severe and fatal pneumonia. Where affordable, clean fuels provide the most certain means of achieving these benefits, but policy needs to actively support this transition. Improved solid fuel stoves may provide some intermediate or even large benefits, but substantial investments are needed in technology and measures to support adoption, and must be accompanied by robust evaluation of longer-term performance, acceptance and health impacts.

## List of abbreviations used

ALRI: acute lower respiratory infection; AQG: air quality guideline; CHERG: Child Health Epidemiology Reference Group; GRADE: Grading of Recommendations, Assessment, Development and Evaluations; HAP: Household air pollution; LBW: low birth weight (less than 2,500 gm); OR: odds ratio; PM2.5: particulate matter with aerodynamic diameter of less than or equal to 2.5 microns; RCT: randomised control trial; WHO: World Health Organisation.

## Competing interests

The authors declare they have no competing interests.

## Funding

Funding for MD, conduct of the evidence review workshop including travel for JD and publication costs are from a grant from the Bill and Melinda Gates Foundation to the US Fund for UNICEF (Grant 43386 to “Promote evidence-based decision making in designing maternal, neonatal and child health interventions in low- and middle-income countries”.

## Authors’ contributions

NB: planning and management of review work, drafting of manuscript.

MD: conduct of searches, data extraction and quality assessment.

JD: conduct of searches, data extraction and quality assessment.

HA-R: Global data on exposure assessment and contributions to systematic reviews.

KB: Methods and data for Indian exposure assessment modeling and contributions to systematic reviews.

ZB: planning and management of review and contributions to systematic reviews.

DP: systematic review, quality assessment and meta-analyses.

All authors read and approved the final manuscript.

## Supplementary Material

Additional file 1 **Search terms and flow charts_HAP review_Bruce** This file lists search terms and databases, and the flow charts to show numbers of studies selected and excluded at each stage. Click here for file

Additional File 2 **GRADE Tabels_HAP review_Bruce** This file shows the GRADE tables for all of the outcomes reviewed. Click here for file

Additional File 3 **Study summary tables_HAP review_Bruce** This file provides summaries of all studies included in the review, listed by outcome. Click here for file

Additional File 4 **Funnel plots__HAP review_Bruce** This file illustrates the funnel plots for two outcomes (i) all pneumonia, and (ii) all-cause mortality. Click here for file

## Declarations

The publication costs for this supplement were funded by a grant from the Bill & Melinda Gates Foundation to the US Fund for UNICEF (grant 43386 to "Promote evidence-based decision making in designing maternal, neonatal, and child health interventions in low- and middle-income countries”). The Supplement Editor is the principal investigator and lead in the development of the Lives Saved Tool (LiST), supported by grant 43386. He declares that he has no competing interests.

This article has been published as part of *BMC Public Health* Volume 13 Supplement 3, 2013: The Lives Saved Tool in 2013: new capabilities and applications. The full contents of the supplement are available online at http://www.biomedcentral.com/bmcpublichealth/supplements/13/S3.
