# Supplementary material for: Control of household air pollution for child survival: estimates for intervention impacts
Source: BMC Public Health. 2013 Sep 17;13(Suppl 3):S8. doi: 10.1186/1471-2458-13-S3-S8 (PMC3847681; doi:10.1186/1471-2458-13-S3-S8)
Supplement: Additional file 1 — Search terms and flow charts_HAP review_Bruce This file lists search terms and databases, and the flow charts to show numbers of studies selected and excluded at each stage. [file 1471-2458-13-S3-S8-S1.docx]

**Additional File 1: Search terms and flowcharts**

**Table A1: Search terms and databases**

**A1(a) Child ALRI**

| **Outcome** | **Exposure** |
| --- | --- |
| 1. “ALRI” 2. “ARI” 3. “pneumonia” 4. “respiratory illness” 5. “respiratory infection” 6. “respiratory disease” 7. “fast breathing” 8. “chest indrawing” “fast breath*” 9. “rapid breath*” 10. “raised respiratory rate” 11. “RSV” 12. “bronchiolitis” 13. “streptococcus pneumoniae” 14. “pneumococcus” 15. “haemophilus influenza” 16. “H. influenza” 17. 1 OR 2 OR 3 OR 4 OR 5 OR 6 OR 7….. OR 16 | 1. “IAP” 2. “Indoor air” 3. “improved stoves” 4. “wood smoke” 5. “dung” 6. “solid fuel” 7. “cooking fuel” 8. “cook* smoke” 9. “stove” 10. “chull*” 11. “heat*” 12. “coal” 13. “pollutant” 14. “pollution” 15. “biomass” 16. “kerosene” 17. “paraffin” 18. 18 OR 19 OR 20 OR 21 OR 22..... OR 34 |
| **Combined terms** | |
| 17. AND 35. | |

**A1(b) Adverse pregnancy outcomes (APOs)**

| **IAP AND LOW BIRTH WEIGHT** | **IAP AND STILLBIRTH** |
| --- | --- |
| *Outcome* | *Outcome* |
| 1. “birth weight” 2. “pre*term” 3. “premature” 4. “small for dates” 5. “growth retardation” 6. “lbw” 7. “1 OR 2 OR 3 OR 4 OR 5 OR 6” | 1. “still*birth” 2. “perinatal mortality” 3. “perinatal death” 4. “1 OR 2 OR 3” |
| *Exposure** |  |
| 1. “biomass” 2. “wood smoke” 3. “indoor air” 4. “iap” 5. “particulate*” 6. “solid fuel” 7. “dung” 8. “cooking fuel” 9. “heating fuel” 10. “coal” 11. “chuj*” 12. “8 OR 9 OR 10 OR 11 OR 12 OR 13 OR 14 OR 15 OR 16 OR 17 OR 18” |  |
| *Combined terms* | *Combined terms* |
| 1. “7 AND 19” | 1. “4 AND 19” |

**A1(c) Stunting and mortality**

| **IAP AND STUNTING** | **IAP AND MORTALITY** |
| --- | --- |
| *Outcome* | *Outcome* |
| 1. “Body height” 2. “Child development” 3. Stunt* 4. “Weight for height” 5. “Height for age” 6. “WHZ” 7. “HAZ” 8. “Growth” | 1. “still*birth” 2. “perinatal mortality” 3. “perinatal death” 4. “child mortality” 5. “infant mortality” 6. “neonatal mortality” 7. “child death” 8. “infant death” 9. “neonatal death” 10. “mortality” |
| *Exposure* |  |
| 1. “biomass” 2. “wood smoke” 3. “indoor air” 4. “iap” 5. “particulate*” 6. “solid fuel” 7. “dung” 8. “cooking fuel” 9. “heating fuel” 10. “coal” 11. “chuj*” 12. “cook smoke” 13. “kerosene” 14. “paraffin” 15. “pollut*” 16. “stove” 17. “HAP” 18. “household air” |  |

**A1(d) Databases:**

**ALRI**

- Published review: Pubmed, Embase, CCRCT, CINAHL, Global Health, DARE, LILACS, SCIELO, Index Medicus Africanus, Chinese CNKI.
- Update 2008-12: Pubmed, Scopus, LILACS, CINHAL, CCRCT, DARE, SIGLE

**Adverse pregnancy outcomes**

- Published review: Medline, EMBASE, CCRCT, CINAHL, LILACS, SIGLE, PASCAL
- Update 2010-12: Scopus, OVID, CINAHL, CCRCT, LILACS

**Stunting and mortality**

- PubMed, EMBASE, MEDLINE,CINAHL, CCT, CSR, ACP, DARE, SIGLE, LILACS

**ALRI searches: 1966 – 2008; 2008 – 2012**

25 + 5 = 30 papers for full data extraction

230 + 35 papers rejected after full review of text

938 + 80 papers rejected on abstracts

255 + 40 full text of papers reviewed

1193 + 120 abstracts reviewed

6212 + 1556 papers identified through searches

5019 + 1436 papers rejected on title

**Birth weight searches 1996 – 2009; 2010 - 2012**

6 (plus 2 unpublished at time of first search) + 5 = 11 papers for full data extraction

3 + 6 papers rejected after full review of text

22 + 67 papers rejected on abstracts

7 + 10 full text of papers reviewed

29 + 77 abstracts reviewed

982+442 papers identified through searches

953 + 375 papers rejected on title

**Still birth Searches 1996 – 2009; 2010 - 2012**

4 (includes 2 unpublished at time of first search) + 1 = 5 papers for full data extraction

1 + 7 papers rejected after full review of text

9 + 37 papers rejected on abstracts

3 + 8 full text of papers reviewed

12 + 45 abstracts reviewed

171 + 151 papers identified through searches

159 + 106 papers rejected on title

**Stunting and mortality: Searches 1996 – 2012**

7 papers for full data extraction (2 stunting; 2 severe stunting; 5 mortality)

16 papers rejected after full review of text

149 papers rejected on abstracts

23 full text of papers reviewed

172 abstracts reviewed

3676 papers identified through searches

3504 papers rejected on title
