# Supplementary material for: Control of household air pollution for child survival: estimates for intervention impacts
Source: BMC Public Health. 2013 Sep 17;13(Suppl 3):S8. doi: 10.1186/1471-2458-13-S3-S8 (PMC3847681; doi:10.1186/1471-2458-13-S3-S8)
Supplement: Additional File 2 — GRADE Tabels_HAP review_Bruce This file shows the GRADE tables for all of the outcomes reviewed. [file 1471-2458-13-S3-S8-S2.docx]

**Additional File 2: GRADE tables**:

All effect estimates are presented as protective effects

**Table 1(a): Non-fatal ALRI**

| Importance of outcome: Important (6) | | | | | | | | | | | |
| --- | --- | --- | --- | --- | --- | --- | --- | --- | --- | --- | --- |
| **Design** | **No. of studies** | **Risk of bias** | **Inconsistency (heterogeneity)** | **Indirectness (external validity)** | **Imprecision (power)** | **Publication bias** | **Other considerations (specify)*** | **Number of events** | | **Relative effect and 95% CI**** | **Quality** |
|  |  |  |  |  |  |  |  | **Intervention** | **Control** |  |  |
| RCTs | 1 | No | No | No | Yes (-1) | No | No | 149 | 180 | 0·78 (0·59–1·06) | MODERATE |
| Observational | 20 | Yes (-1) | Yes (-1) | No | No | Possible (-1) | No | 11,331 total events | | 0.63 (0.53, 0.75) | VERY LOW |

*Only large effect is used for upgrading if the group of studies is downgraded for any reason

**Relative effects are presented as originally published, that is as a protective RR for the intervention in the RCT, but as risk of ‘exposed’ vs. ‘non-exposed’ for observational studies

**Table 1(b): Severe ALRI**

| Importance of outcome: Critical (9) | | | | | | | | | | | |
| --- | --- | --- | --- | --- | --- | --- | --- | --- | --- | --- | --- |
| **Design** | **No. of studies** | **Risk of bias** | **Inconsistency (heterogeneity)** | **Indirectness (external validity)** | **Imprecision (power)** | **Publication bias** | **Other considerations (specify)*** | **Number of events** | | **Relative effect and 95% CI**** | **Quality** |
|  |  |  |  |  |  |  |  | **Intervention** | **Control** |  |  |
| RCTs | 1 | No | No | No | No | No | Exposure-response (+1) | 72 | 101 | 0·67 (0·45–0·98) | HIGH |
| Observational | 3 | Yes (-1) | No | No | No | No | Large effect (+1) | 331 events | | 0.40 (0.25, 0.67) | LOW |

**Table 1(c): Fatal ALRI**

| Importance of outcome: Critical (9) | | | | | | | | | | | |
| --- | --- | --- | --- | --- | --- | --- | --- | --- | --- | --- | --- |
| **Design** | **No. of studies** | **Risk of bias** | **Inconsistency (heterogeneity)** | **Indirectness (external validity)** | **Imprecision (power)** | **Publication bias** | **Other considerations (specify)*** | **Number of events**** | | **Relative effect and 95% CI**** | **Quality** |
|  |  |  |  |  |  |  |  | **Intervention** | **Control** |  |  |
| RCTs | 1 | No | No | No | Yes (-1) | No | Large effect (+1) | 3 | 6 | 0.48 (0.12, 1.91) | [HIGH] |
| Observational | 3 | Yes (-1) | No | No | No | No | Large effect (+1) | 659 events | | 0.34 (0.22, 0.55) | LOW |

**Table 2: Low birth weight**

| Importance of outcome: Important (6) | | | | | | | | | | | |
| --- | --- | --- | --- | --- | --- | --- | --- | --- | --- | --- | --- |
| **Design** | **No. of studies** | **Risk of bias** | **Inconsistency (heterogeneity)** | **Indirectness (external validity)** | **Imprecision (power)** | **Publication bias** | **Other considerations (specify)*** | **Number of events** | | **Relative effect and 95% CI**** | **Quality** |
|  |  |  |  |  |  |  |  | **Intervention** | **Control** |  |  |
| RCTs | 1 | No | No | No | Yes (-1) | No | No | 13 | 26 | 0.74 (0.33-1.66) | MODERATE |
| Observational | 6 | No | No | No | No | No | No | 5670 events | | 0.71 (0.64, 0.79) | LOW |

**Table 3: Stillbirth**

| Importance of outcome: Critical (9) | | | | | | | | | | | |
| --- | --- | --- | --- | --- | --- | --- | --- | --- | --- | --- | --- |
| **Design** | **No. of studies** | **Risk of bias** | **Inconsistency (heterogeneity)** | **Indirectness (external validity)** | **Imprecision (power)** | **Publication bias** | **Other considerations (specify)*** | **Number of events** | | **Relative effect and 95% CI** | **Quality** |
|  |  |  |  |  |  |  |  | **Intervention** | **Control** |  |  |
| Observational | 4 | No | No | No | No | No | No | 3345 events | | 0.66 (0.54, 0.81) | LOW |

**Table 4: Pre-term birth**

| Importance of outcome: Important (6) | | | | | | | | | | | |
| --- | --- | --- | --- | --- | --- | --- | --- | --- | --- | --- | --- |
| **Design** | **No. of studies** | **Risk of bias** | **Inconsistency (heterogeneity)** | **Indirectness (external validity)** | **Imprecision (power)** | **Publication bias** | **Other considerations (specify)*** | **Number of events** | | **Relative effect and 95% CI** | **Quality** |
|  |  |  |  |  |  |  |  | **Intervention** | **Control** |  |  |
| Observational | 1 | No | No | No | No | No | No | 1568 events | | 0.70 (0.54, 0.90) | LOW |

**Table 5: Stunting (all observational designs)**

| Importance of outcome: Important (6) | | | | | | | | | | | |
| --- | --- | --- | --- | --- | --- | --- | --- | --- | --- | --- | --- |
| **Design** | **No. of studies** | **Risk of bias** | **Inconsistency (heterogeneity)** | **Indirectness (external validity)** | **Imprecision (power)** | **Publication bias** | **Other considerations (specify)*** | **Number of events** | | **Relative effect and 95% CI** | **Quality** |
|  |  |  |  |  |  |  |  | **Intervention** | **Control** |  |  |
| Stunting | 2 | No | No | No | No | No | No | 7109 events | | 0.79 (0.70, 0.89) | LOW |
| Severe stunting | 2 | No | Yes (-1) | No | No | No | No | 8157 events | | 0.64 (0.43, 0.96) | VERY LOW |

**Table 6: All cause mortality**

| Importance of outcome: Critical (9) | | | | | | | | | | | |
| --- | --- | --- | --- | --- | --- | --- | --- | --- | --- | --- | --- |
| **Design** | **No. of studies** | **Risk of bias** | **Inconsistency (heterogeneity)** | **Indirectness (external validity)** | **Imprecision (power)** | **Publication bias** | **Other considerations (specify)*** | **Number of events** | | **Relative effect and 95% CI** | **Quality** |
|  |  |  |  |  |  |  |  | **Intervention** | **Control** |  |  |
| Observational | 5 | No | Yes (-1) | No | No | Possible | No | 8446 events | | 0.79 (0.70, 0.89) | VERY LOW |
