# Supplementary material for: Control of household air pollution for child survival: estimates for intervention impacts
Source: BMC Public Health. 2013 Sep 17;13(Suppl 3):S8. doi: 10.1186/1471-2458-13-S3-S8 (PMC3847681; doi:10.1186/1471-2458-13-S3-S8)
Supplement: Additional File 3 — Study summary tables_HAP review_Bruce This file provides summaries of all studies included in the review, listed by outcome. [file 1471-2458-13-S3-S8-S3.docx]

**Additional File 3: Study summary tables**

**Table 1. Child pneumonia/ALRI**

***(a) Non-fatal ALRI with severity not defined***

| **Study identifier and setting** | **Study design, age and sample size** | **Intervention or exposure assessment** | **Outcome assessment** | **Main results and numbers of events (from prevalence where relevant)** | **Summary of limitations, including quality assessment score** |
| --- | --- | --- | --- | --- | --- |
| **A. Randomized trials** | | | | | |
| Smith (2011)  Rural Guatemala | Individual (not cluster) RCT with partial blinding; 265 intervention and 263 control children < 19 months. | Improved chimney stoves vs. traditional open fire; individual exposure assessment. | Physician diagnosed pneumonia. | Child exposure reduced by 50%; 149 (intervention) and 180 (control) cases. RR=0.78 (0.59, 1.06) p=0.095 | QA score = 11.5/14  Post intervention exposure still high; Main outcome NS (p=0.095), significant (adjusted) exposure-response |
| **B. Cross-sectional studies** | | | | | |
| Mishra 2003  Urban and rural Zimbabwe | 1999 DHS sample; 3,559 children < 60 months. | Interview survey for main cooking fuel type, 66% used biomass. | Mother’s recall of ALRI symptoms and signs in prior 2 weeks | 563 (approx) cases; Adjusted OR biomass vs. clean fuel: 2.20 (1.16, 4.19) | QA score = 5/13. Not adjusted for ETS and breastfeeding |
| Mishra 2005  Urban and rural India | 1989-90 National Family Health Survey; 29,768 children < 36 months. | Interview survey for main cooking fuel type, 64% used biomass. | Mother’s recall of ALRI symptoms and signs in prior 2 weeks | 5834 (approx) cases; Adjusted OR biomass vs. clean fuel: 1.58 (1.28, 1.95) | QA score = 5.5/13. Not adjusted for vaccination and breastfeeding |
| Wichmann 2006  Urban and rural South Africa | 1998 DHS; 4,679 children < 60 months. | Interview survey for main cooking fuel type, 39% used biomass. | Mother’s recall of ALRI symptoms and signs in prior 2 weeks | 900 (approx) cases; Adjusted OR biomass vs. clean fuel: 1.29 (1.02, 1.62) | QA score = 5.5/13. Not adjusted for vaccination, ETS, breastfeeding, nutritional status. |
| Kashima 2010  Urban and rural Indonesia | Indonesian DHS; 15,242 singleton births | Interview on cooking fuel type, % used solid fuels. Solid fuel used by 71% (rural) and 13% urban. Kerosene included in clean fuel group. | Interview on symptoms and signs (fast breathing) of ALRI in prior 2 weeks. | 462 (urban) and 801 (rural) cases; adjusted OR for solid fuel vs. clean: 0.99 (0.67, 1.30) urban; 1.09 (0.89, 1.34) rural | QA score = 4/13. Clean fuel category includes kerosene. Limited adjustment for SES, and not for ETS and nutrition. |
| **C. Case-control studies** | | | | | |
| Kossove 1982  Urban South Africa | 132 cases (outpatient) and 18 controls (outpatient, non-respiratory illness), age <13 months | Interview asking if child stays in cook smoke, 33% exposed. | Physician diagnosis with chest X-ray | 130 cases; unadjusted OR exposed vs. unexposed: 4.77 (1.51, 16.46) | QA score = 3.5/12. Small control group, vague exposure assessment, inadequate adjustment. |
| Collings 1990  Urban and rural Zimbabwe | 244 cases (hospital) and 500 controls (well baby) sampled from hospital, age 1-36 months. | Interview on exposure to wood smoke or clean fuel; measurement in 40 homes, 73% used biomass. | Physician diagnosed with chest X-ray | 244 cases; unadjusted OR for wood fire vs. clean fuel: 2.14 (1.41, 3.36). | QA score = 4.5/12. Possible control bias, unadjusted. |
| Morris 1990  Rural Arizona, USA. | 58 cases (outpatient) and 58 controls (well children, same clinic), age 2 weeks to <24 months. | Interview on primary source of heating and cooking (fuel type), 57% used wood for heating. | X-ray confirmed pneumonia, and bronchiolitis. | 58 cases; adjusted OR for wood stove vs. no use of wood stove: 4.85 (1.69, 12.91). | QA score = 5/12. Possible control bias, combine pneumonia and bronchiolitis. |
| Johnson 1992  Urban Nigeria | 103 cases (inpatient) and 103 controls (well baby) from hospital, age 2 weeks to <60 months. | Interview on cooking fuel and location of cooking rate(separate from or within living/sleeping area), 19% used biomass. | Physician diagnosis with chest X-ray. | 103 cases; unadjusted OR for firewood vs. petroleum products 0.80 (0.36, 1.78); location: 1.45 (0.75, 2.81) | QA score = 4/12. Possible control bias, unadjusted. |
| Victora 1994  Urban Brazil | 510 cases (inpatient) and 510 controls (community), age <24 months. | Interview on indoor smoke from cooking and other sources, 6% exposed. | Admitted with X-ray confirmed pneumonia | 510 cases; unadjusted OR for exposed to indoor smoke (comparison not described): 1.1 (0.61, 1.98) | QA score = 7.5/12. Well conducted but unadjusted, poor exposure assessment |
| Azizi 1995  Urban Malaysia | 271 cases (inpatient) and 322 control (inpatient, non-respiratory illness), age 1 – 60 months. | Interview on cooking fuel use, prevalence of wood use, 6.8% used biomass. | Physician diagnosed pneumonia (X-ray), bronchiolitis, empyema. | 271 cases; unadjusted OR for wood fuel vs. cleaner fuel: 1.20 (0.65, 2.21) | QA score 7.5/12. Adjusted estimates obtained but not reported. |
| Robin 1996  Rural Arizona, USA. | 45 cases (inpatient) and 45 controls (cardio-respiratory outpatients, same hospital), age 1 – 24 months. | Interview on heating and cooking fuel, 51% used wood for heating. Also measured 15-hr PM2.5 in all homes. | Physician diagnosed pneumonia and bronchiolitis. | 45 cases; unadjusted OR for wood (alone) for heating vs. clean fuel: 1.4 (0.6, 3.3). | QA score 7.5/12. Unadjusted analysis, but demonstrated PM2.5 to be higher in wood-using homes. |
| Fonseca 1996  Urban Brazil | 650 cases (hospital) and 650 controls (community), age < 24 months. | Interview on indoor smoke from poor cooking facilities or lighting, 10% exposed. | Radiologically confirmed pneumonia | 650 cases. Adjusted OR for indoor smoke vs. not: 1.14 (0.71, 1.81) | QA score 8/12. Well conducted study but with poor exposure assessment. |
| O’Dempsey 1996  Rural Gambia | 80 cases (outpatient, pneumococcal disease) and 159 controls community), age <60 months. | Interview on whether mother carries child while cooking, 44% exposed. | Clinical and laboratory confirmed evidence of pneumococcal infection. | 80 cases; adjusted OR for carried vs. not carried: 2.55 (0.98, 6.65) | QA score 8/12; used community controls, exposure measure uncertain |
| Wesley 1996  Peri-urban South Africa | 48 cases (inpatient) and 48 controls (AURI same hospital), age 3 – 36 months. | Interview on fuel used for cooking and heating, 14% used wood or coal. | Physician diagnosed pneumonia | 48 cases; unadjusted OR for wood/coal (comparison not stated, presume clean fuel): 1.35 (0.40, 4.71) | QA score = 4/12. Control selection may be biased, poor exposure assessment, unadjusted. |
| Mahalanabis 2002  Urban India | 127 cases (inpatients) and 135 controls (immunization clinic), age 2 – 35 months. | Information obtained (method not described) on fuel, 29% used some solid fuels. | Physician diagnosis of pneumonia and bronchiolitis | 127 cases; adjusted OR for solid fuel (comparison not stated, presume clean fuel): 3.97 (2.00, 7.88). | QA score: 5.3/12. Combines pneumonia and bronchiolitis, possible control bias, poor exposure assessment. |
| **D. Cohort studies** | | | | | |
| Pandey 1989  Rural Nepal (Study I) | 780 children, followed for 6 months; second study excluded due to very high OR. | Interview on average hours per day child near fireplace, 55% exposed >2 hours/day. | Community assessed pneumonia based on symptoms and signs | 63 cases; unadjusted OR for exposure > 2hours vs. < 2 hours: 2.2 (1.6, 3.0) | QA score = 4/13. Unadjusted, exposure-response relationship reported. |
| Campbell 1989  Rural Gambia | 280 children, age <24 months | Interview on whether child carried on the mother’s back while cooking, % not reported. | Parental recall of fast breathing at weekly visits | Cases not reported; adjusted OR for carried on back vs. not carried: 2.80 (1.29, 6.09) | QA score = 3/13. Uncertain exposure assessment, authors cautious about interpretation. |
| Armstrong 1991  Rural Gambia | 587 children, age <60 months followed for 3 months. | Interview on whether child carried on the mother’s back while cooking, 37% carried. | Identified through weekly home visits, but clinical and chest X-ray signs required. | 75 cases; adjusted OR (all episodes) for carried vs. not carried: 0.5 (0.2, 1.3) males; 1.9 (1.0, 3.9) females. | QA score = 7/13. Uncertain exposure assessment, male vs. female difference may be due to lower exposure (boys). |
| Ezzati 2001  Rural Kenya | 93 children from 55 homes, age <60 months | Combination of micro-environment measurement of PM_10_ and time-activity. | Bi-weekly home visits to identify children with WHO ALRI symptoms and signs. | Cases not stated; adjusted OR for mid-value exposure level (1-2,000 µg/m^3^) vs. lowest (<200 µg/m^3^): 2.33 (1.23, 4.38). | QA score = 7.5/13. Adjustment did not include breastfeeding, vaccination, ETS. |
| Bautista 2009  Urban Dominican Republic | 415 children aged <18 months followed for 1 year/until 2 years of age. | Interview on fuel used for cooking; measurement of PM (respirable) on sub-sample. | Bi-weekly home visit, with cases confirmed by physician diagnosis | 367 cases; adjusted OR for charcoal vs. propane: 1.38 (1.06, 1.81) | QA score = 10/13. Measured exposure supports analysis, but sample not described. |

***(b) Severe pneumonia***

| **Study identifier and setting** | **Study design, age and sample size** | **Intervention or exposure assessment** | **Outcome assessment** | **Main results and numbers of events (from prevalence where relevant)** | **Summary of limitations, including quality assessment score** |
| --- | --- | --- | --- | --- | --- |
| **A. Randomized trials** | | | | | |
| Smith 2011  Rural Guatemala | Individual (not cluster) RCT with partial blinding; 265 intervention and 263 control children < 19 months. | Improved chimney stoves vs. traditional open fire; individual exposure assessment. | Physician diagnosed pneumonia, pulse oximetry used to define severe outcome. | Child exposure reduced by 50%; 72 (intervention) and 101 (control) cases: RR=0.67 (0.45, 0.98). | QA score = 11.5/14  Post intervention exposure still high; significant exposure-response |
| **B. Observational studies** | | | | | |
| Wayse 2004  Urban India | Case-control study; 80 cases (hospital) and 70 controls (attending immunization clinic), age <60 months. | Interview on cooking fuels, 43% used biomass. | Physician diagnosis of severe ALRI | 80 cases; adjusted OR for biomass vs. LPG: 1.39 (0.57, 3.22) | QA score = 5.5/12. Control bias possible, limited description for exposure (fuel use). |
| Broor 2001  Urban and rural India | Case-control study; 201 cases (hospital) and 311 control (well children attending immunization clinic), age <50 months. | Interview on type of cooking fuel, 21% used fuel ‘other than LPG’. | Physician diagnosed cases, with requirement for fast breathing and lower chest wall indrawing. | 201 cases; adjusted OR for exposure to fuel ‘other than LPG’ vs. LPG: 2.51 (1.51, 4.16). | QA score = 5.5/12. Exposure categories poorly described, control bias possible. |
| Kumar 2004  Urban and rural India | Case-control study; 50 cases (inpatient) and 50 controls (immunization clinic), age 2 to <60 months. | Interview on type of cooking fuel, LPG or other (coal, biomass and kerosene), 36% used fuel other than LPG. | Unclear, but presume physician diagnosis, and ‘satisfying criteria for severe pneumonia’. | 50 cases; adjusted OR for fuel ‘other than LPG’ vs. LPG: 3.87 (1.15, 12.43). | QA score = 4/12. Control bias possible, breast feeding and crowding not adjusted for. |

***(c) Fatal pneumonia***

| **Study identifier and setting** | **Study design, age and sample size** | **Intervention or exposure assessment** | **Outcome assessment** | **Main results and numbers of events (from prevalence where relevant)** | **Summary of limitations, including quality assessment score** |
| --- | --- | --- | --- | --- | --- |
| **A. Randomized trials** | | | | | |
| Smith 2011  Rural Guatemala | Individual (not cluster) RCT with partial blinding; 265 intervention and 263 control children < 19 months. | Improved chimney stoves vs. traditional open fire; individual exposure assessment. | Physician diagnosed pneumonia, deaths ascertained by verbal autopsy. | Child exposure reduced by 50%; 3 (intervention) and 6 (control) deaths: RR=0.48 (0.12, 1.91). | QA score = 11.5/14  Post intervention exposure still high; significant (adjusted) exposure-response. |
| **B. Observational studies** | | | | | |
| De Francisco 1993  Rural Gambia | Case-control study; 129 cases and 270 live controls (community), 144 dead controls (other causes). | Interview on type and location of cooking stove, carrying child while cooking, prevalence not reported. | Deaths ascertained by community surveillance, cause determined by verbal autopsy. | 129 deaths; adjusted OR using live controls for always carrying child vs. never carrying: 5.23 (1.72, 15.92) | QA score 6.5/12. Uncertain exposure measure, live (community) controls should avoid bias. |
| Johnson 2008  Urban Nigeria | Case-fatality study; age 2-59 months, followed for 30 months. | Interview on type of cooking fuel, proximity of cooking area to living/sleeping rooms, 17% use wood or kerosene. | Clinical diagnosis with radiological confirmation. | 323 deaths; unadjusted OR for wood or kerosene vs. electricity or gas: 2.92 (1.32, 6.50). | QA score = 7/13. Unadjusted effect estimate, exposed category includes kerosene. |
| Rehfuess 2009  16 African countries | Cross-sectional study; 32, 620 children in World Health Survey, dying before 5 years | Interview on cooking fuel type, location and ventilation, average 87% used solid fuels. | Interview on death (within 10 years) and associated symptoms and signs. | 207 deaths; adjusted hazard ratio for solid fuel vs. cleaner fuel: 2.35 (1.22, 4.52). | QA score = 6/13. Uncertain validity of cause of death based on long-term recall. |

**Table 2: Low birth weight**

| **Study identifier and setting** | **Study design, age and sample size** | **Intervention or exposure assessment** | **Outcome assessment** | **Main results and numbers of events (from prevalence where relevant)** | **Summary of limitations, including quality assessment score** |
| --- | --- | --- | --- | --- | --- |
| **A. Randomized trials** | | | | | |
| Thompson (2005)  Rural Guatemala | 225 singleton births (104 intervention and 121 control). | Improved chimney stoves vs. traditional open fire; individual exposure assessment | Birth weight using scales by trained field worker (67% within 48 hrs). LBW = <2500g. | 50 cases of LBW (22%); adjusted RR for chimney stove vs. open fire: 1.30 (0.72, 2.35). | QA Score = 8/13. Analysis per protocol due to delay in intervention distribution. |
| **B. Observational studies** | | | | | |
| Boy (2002)  Rural Guatemala | Cross-sectional survey. Singleton births: Home (field worker - 572) and public hospital (all - 1145). | Interview. Cooking with wood/coal vs. gas/ electricity. 871 (71%) exposed (wood/coal with no chimney). | Birth weight using scales: trained field worker for home (majority in 72 hrs) and nurses for hospital (all in 24 hrs). LBW = <2500g. | 230 cases of LBW (19%); unadjusted OR for wood/coal vs. clean fuel: 1.30 (CI=0.92, 1.83). | QA Score = 9/11. Unadjusted analysis for % LBW (adjusted analysis for mean birth weight available). |
| Mavalankar (1992)  India | Case-control study. Singleton births: 1731 LBW (<2,500g) and 1465 controls (>2500g). | Self reported “exposure to cook fire smoke. 442 (30%) of control mothers exposed. | Physician measured outcome LBW (birth weighing not specified). | 1731 cases of LBW (case-control); unadjusted OR for exposed: **Term:** 1.23 (1.01, 1.5);  **Preterm:** 1.49 (1.22, 1.82). | QA Score = 6/10. Unadjusted analysis. |
| Mishra (2004)  Zimbabwe | Cross-sectional survey. National 2-stage cluster sample of 2610 singleton births (75% in health facility). | Interview – main cooking fuel: high pollution (biomass) vs. low (gas/ electricity). 1228 (47%) exposed. | Birth weight from health cards (1390) and mother’s recall (1220). | 191 cases of LBW (8%); unadjusted OR for biomass vs. clean fuel: 1.12 (0.80, 1.56). | QA Score = 6/11. Uncertain validity of maternal recall of birth weight, unadjusted analysis. |
| Siddiqui (2008)  Pakistan | Cohort study. 634 pregnant women recruited from health surveillance program. Singleton births only. | Interview – main cooking fuel:  wood vs. gas. 366 (58%) using wood. | Birth weight using scales: Trained fieldworkers (91% within 24 hours). | 123 cases of LBW (19%); adjusted OR for wood vs. LPG: 1.77 (1.09, 2.88). | QA Score = 7/10. |
| Tielsch (2009)  India | Cohort study. 11,728 live born infants (not reported whether singleton). | Interview – main cooking fuel:  wood/dung vs. gas/kerosene, 8958 (93%) using wood/ dung for cooking. | Birth weight using scales: Trained fieldworkers (82% (9604) within 72 hours – others excluded). | 3172 cases of LBW (27%); adjusted OR for biomass vs. LPG/kerosene: **Term:** 1.49 (1.25, 1.77). **Preterm:** 1.70 (0.93, 3.10). | QA Score = 7/10. Clean fuel category includes kerosene. |
| Abusalah (2011)  Gaza Strip | Matched case-control study. 2 hospitals. Singleton births: 223 cases (<2500g) and 223 matched controls (>=2500g). | Interview – within 24 hours from delivery. Self-reported exposure to wood fuel smoke. 24 (11%) of control mothers exposed. | Birth weight using scales by trained qualified nurses. | 223 cases of LBW (case-control); adjusted OR for self reported wood smoke exposure : 2.3 (1.2, 4.7). | QA Score = 6/10. |

**Table 3: Stillbirth**

All available studies are observational

| **Study identifier and setting** | **Study design, age and sample size** | **Intervention or exposure assessment** | **Outcome assessment** | **Main results and numbers of events (from prevalence where relevant)** | **Summary of limitations, including quality assessment score** |
| --- | --- | --- | --- | --- | --- |
| Mavalankar (1991)  India | Case-Control study. Singleton births from 3 hospitals: 451 still births and 160 early neonatal deaths (cases) and 1465 controls (live births up to 7 days). | Self-reported “exposure to cooking smoke”. 442 (30%) of control mothers exposed. | Trained physician. Stillbirth and early neonatal deaths (with 1 week) in hospital. | 451 stillbirths (case-control); adjusted OR for exposed: 1.50 (1.00, 2.10). | QA Score = 6/10. |
| Mishra (2005)  Urban and rural India | Cross-sectional survey. Nationally representative sample of 19189 women. singleton births only. | Interview – main cooking fuel: high pollution (biomass) vs. low (gas/ electricity). | Stillbirth = delivery of a dead baby after 28 weeks pregnancy. | 2451 stillbirths (12%); adjusted OR for biomass vs. clean fuel: 1.44 (1.04, 1.97). | QA Score = 7/11. |
| Siddiqui (2005)  Urban Pakistan | Cohort study. 1102 pregnant women recruited from health surveillance program. Singleton births only. | Interview – main cooking fuel:  wood vs. gas. 584 (53%) using wood for cooking. | Reproductive loss through stillbirth (n=85). | 85 stillbirths (8%); adjusted OR for cooking with wood vs. gas: 1.90 (1.10, 3.20). | QA Score = 5/10. |
| Tielsch (2009)  Rural India | Cohort study. 11,728 live born infants (not reported whether singleton). | Interview – main cooking fuel:  wood/dung vs. gas/kerosene, 8958 (93%) using wood/dung for cooking.. | Trained interviewers. Delivery within 28 weeks in which the fetus was born dead (n=358). | 358 stillbirths (3%); adjusted OR for cooking with biomass vs. cleaner fuel/kerosene): 1.34 (0.76, 2.36). | QA Score = 7/10. Clean fuel includes kerosene. |

**Table 4: Pre-term birth**

All available studies are observational

| **Study identifier and setting** | **Study design, age and sample size** | **Intervention or exposure assessment** | **Outcome assessment** | **Main results and numbers of events (from prevalence where relevant)** | **Summary of limitations, including quality assessment score** |
| --- | --- | --- | --- | --- | --- |
| Tielsch (2009)  Rural India | Cohort study. 11,728 live born infants (not reported whether singleton). | Interview – main cooking fuel:  wood/dung vs. gas/kerosene, 8958 (93%) using wood/ dung for cooking. | Prematurity defined as < 37 weeks gestation. | 1568 pre-term births (14%); adjusted OR for cooking with biomass vs. kerosene/LPG: 1.43 (1.11, 1.84). | QA Score = 7/10. Clean fuel includes kerosene. |

**Table 5: Stunting (moderate and severe)**

All available studies are observational

| **Study identifier and setting** | **Study design, age and sample size** | **Intervention or exposure assessment** | **Outcome assessment** | **Main results and numbers of events (from prevalence where relevant)** | **Summary of limitations, including quality assessment score** |
| --- | --- | --- | --- | --- | --- |
| **Moderate stunting** | | | | | |
| Kyu (2009)  Urban and rural settings | Cross-sectional study, based on DHS surveys in 7 countries (Cambodia, Dominican Republic, Haiti, Jordan, Moldova, Namibia, Nepal); 28,439 children < 5 years. | Interview on fuel used for cooking, biofuels, mixed and clean (includes kerosene), 51% used biofuels. | Height measured during survey. | 4209 (approx) cases of stunting; adjusted OR for biomass vs. clean fuels (includes kerosene): 1.25 (1.08, 1.44). | QA score = 4/13. Clean fuel includes kerosene. Countries included have widely differing exposure to biomass and nutritional status. |
| Tielsch ( 2009)  Rural India | Cohort study; 10,437 infants followed to 6 months of age. | Interview – main cooking fuel:  wood/dung vs. gas/kerosene, 93% used biomass.  . | Bi-weekly home visits for measurement of weight and height | 2900 (approx) cases of stunting; adjusted OR for cooking with biomass vs. kerosene/LPG: 1.30 (1.06, 1.60). | QA Score = 6/13. Clean fuel includes kerosene. |
| **Severe stunting** | | | | | |
| Mishra (2007)  Urban and rural India | Cross sectional study, based on 1998-99 National Family Health Survey. 29,768 children aged <36 months. | Interview on fuel used for cooking, biofuels, mixed and clean (includes kerosene), 64% used biofuels. | Height measured during survey. | 5882 cases of severe stunting; adjusted OR for cooking with biomass vs. clean fuels: 1.90 (1.49, 2.42). [Adjusted OR for moderate stunting = 1.25 p<0.05] | QA Score = 4/13. Clean fuel includes kerosene. Estimates for stunting also provided but without 95% CI or exact p-value, so not included in meta-analysis. |
| Kyu (2009) | Cross-sectional study, based on DHS surveys in 7 countries (see above); 28,439 children < 5 years. | Interview on fuel used for cooking, biofuels, mixed and clean (includes kerosene), 51% used biofuels. | Height measured during survey. | 2275 (approx) cases of severe stunting; adjusted OR for biomass vs. clean fuels (includes kerosene): 1.27 (1.02, 1.59). | QA score = 4/13. Clean fuel includes kerosene. Countries included have widely differing exposure to biomass and nutritional status. |

**Table 6: All-cause mortality**

All available studies are observational

| **Study identifier and setting** | **Study design, age and sample size** | **Intervention or exposure assessment** | **Outcome assessment** | **Main results and numbers of events (from prevalence where relevant)** | **Summary of limitations, including quality assessment score** |
| --- | --- | --- | --- | --- | --- |
| Mtango (1992)  Rural Tanzania | Case-control study; 610 cases and 1160 living controls (community). | Interview on type of cooking fuel and whether child sleeps in cooking area, 95% controls used wood for cooking. | Deaths ascertained by community surveillance, cause by health records and verbal autopsy*. | 610 deaths; adjusted OR for sleeping in room with cooking vs. not doing so: 2.78 (1.79, 4.33) | QA score 5/12. Staff collecting data differed between cases and controls. |
| Wichmann (2006)  Urban and rural South Africa | Cross-sectional study, based on DHS; 3556 children aged <60 months | Interview on cooking and heating fuel (solid fuel and kerosene, compared to gas and electricity), 79% used solid fuel/kerosene. | Interview on occurrence of death in children <5 years (at any time). | 142 deaths; adjusted RR for solid fuel/kerosene vs. clean fuel: 1.95 (1.04, 3.68). | QA score 6/13. Adjustment does not include ETS and vaccination. Inconsistency between text and Table 2 (OR=1.99, same 95% CI) |
| Tielsch (2009)  Rural India | Cohort study; 10,437 infants followed to 6 months of age. | Interview on type of main cooking fuel: wood/dung vs. gas/kerosene, 93% used biomass. | Bi-weekly home visits to 6 months of age, deaths recorded. | 369 neonatal deaths; adjusted RR for biomass vs. gas/kerosene: 1.17 (0.70, 1.96).  586 deaths 0-6 months; adjusted RR for biomass vs. gas/kerosene: 1.21 (0.79, 1.84). | QA score 8/13. Clean fuel category includes kerosene. |
| Bassani (2010)  Urban and rural India | Case-control study; Special Fertility and Mortality Survey (India); 6790 deaths and 609,601 living children, age < 5 years. | Interview on cooking fuel, categorized as solid fuel and non-solid (includes kerosene), 77% used solid fuels. | All deaths under age 5 years during 1997, stratified into post-neonatal, and deaths aged 1 to <5 years. | **Post-neonatal:** 1436 (male) and 1790 (female) deaths: adjusted OR for solid fuels vs. non-solid: 0.95 (0.79, 1.15) male; 0.91 (0.77, 1.07) female.  **Aged 1 to <5 years:** 1568 (male) and 1996 (female) deaths: adjusted OR for solid fuel vs. non-solid: 1.30 (1.08, 1.56) male; 1.33 (1.12, 1.58) female. | QA score = 5.5/12. Unknown proportion of kerosene using homes included in non-solid fuel group; no adjustment for breast feeding or nutritional status. For deaths aged 1-<5 years, significant exposure-response trends were reported across non-separate and separate kitchen for solid fuel. |
| Kashima (2010)  Urban and rural Indonesia | Indonesian DHS; 15,242 singleton births, age < 5 years, deaths in neonatal period and infancy (<12 months). | Interview on cooking fuel type, % used solid fuels. Solid fuel used by 71% (rural) and 13% urban. Kerosene included in clean fuel group. | Interview on death in neonatal period (neonatal mortality), and in first year of life (infant mortality). Assessment of stillbirth combined with miscarriage and abortion, which does not meet inclusion criteria. | **Neonatal**: 103 (urban) and 182 (rural) deaths; adjusted OR for solid fuel vs. clean: 1.29 (0.75, 2.24) urban; 1.05 (0.72, 1.54) rural.  **Infant**: 153 (urban) and 382 (rural) deaths; adjusted OR for solid fuel vs. clean: 1.47 (0.92, 2.33) urban; 1.31 (1.00, 1.70). | QA score = 4/13. Clean fuel category includes kerosene. Limited adjustment for SES, and not for ETS and nutrition. Adjust for outdoor air pollution using (crude) road proximity. |

*The Mtango 1992 study was not included in cause-specific analysis for fatal pneumonia as neither the 95% CI nor the SE are provided (adjusted OR for same exposure comparison = 4.29)
