# Supplementary material for: Control of household air pollution for child survival: estimates for intervention impacts
Source: BMC Public Health. 2013 Sep 17;13(Suppl 3):S8. doi: 10.1186/1471-2458-13-S3-S8 (PMC3847681; doi:10.1186/1471-2458-13-S3-S8)
Supplement: Additional File 4 — Funnel plots__HAP review_Bruce This file illustrates the funnel plots for two outcomes (i) all pneumonia, and (ii) all-cause mortality. [file 1471-2458-13-S3-S8-S4.docx]

**Additional File 4: Funnel plots**

**Figure 1**: Funnel plot for studies of non-fatal ALRI, where severity is not specified

**Figure 2**: Funnel plot for studies of all-cause mortality
